# Atrial fibrillation in aortic stenosis - echocardiographic assessment and prognostic importance

**DOI:** 10.1186/1476-7120-10-38

**Published:** 2012-09-25

**Authors:** Charlotte Burup Kristensen, Jan Skov Jensen, Peter Sogaard, Helle Gervig Carstensen, Rasmus Mogelvang

**Affiliations:** 1 Copenhagen University Hospital Gentofte, Department of Cardiology P835, Niels Andersens vej 65, DK-2900, Hellerup, Denmark; 2 Copenhagen University Hospital Rigshospitalet, Department of Cardiology B2142, Blegdamsvej 9, DK-2100, Copenhagen Ø, Denmark

**Keywords:** Aortic stenosis, Atrial fibrillation, Echocardiography, Mortality

## Abstract

**Background:**

Atrial fibrillation (AFib) exists more frequently in patients with aortic stenosis (AS) than in patients without, and AFib may be a sign of progressive deterioration of AS. Echocardiographic assessment of AS in sinus rhythm is well documented, however, little is known about AFib in AS since such patients often are excluded from clinical echocardiographic trials.

**Aim:**

The purpose of this study was to assess the prognostic importance of AFib in AS.

**Methods:**

The study was designed as a single-center case-control study. Patients with AS and AFib were enrolled as cases (n = 103) and subsequently matched to controls (103 patients with AS but sinus rhythm). Cases and controls were matched according to age, gender and severity of AS. Primary outcome was all cause mortality and follow-up was 100% complete.

**Results:**

Compared to controls the group with AFib had lower mean ejection fraction (42% vs. 49%; p < 0.001) and stroke volume (47 mL vs. 55 mL; p = 0.004), but higher heart rate (81 bpm vs. 68 bpm; p < 0.001) and no significant difference with regard to cardiac output (3.8 L vs. 4.0 L; p = 0.29). Accordingly, aortic jet velocity and gradients were significantly lower in AFib compared to controls but there were no differences (p = 0.38) in aortic valve area calculated by the continuity equation. During a median follow-up of 2.3 years (IQR: 1.2-3.6), 70 (34%) patients with AS died: 42 patients with AFib and 28 patients with sinus rhythm (p < 0.02). After adjusting for echocardiographic significant differences, AFib remained an independent predictor of mortality (HR 2.72 (95% CI: 1.12–6.61), p < 0.03). There was no significant interaction (p = 0.62) between AFib and AS on the risk of mortality, indicating that AFib predicted bad outcome regardless of the severity of AS.

**Conclusions:**

AFib is an independent risk factor in patients with AS and the prognostic impact of AFib seems to be the same despite the severity of AS.

## Background

Aortic stenosis (AS) and atrial fibrillation (AFib) are two conditions associated with high cardiovascular morbidity and mortality [1-5]. The incidences increase with age and as populations are ageing; both conditions are likely to become a greater public burden [1,2,6].

The left ventricular (LV) response to AS is hypertrophy, impaired relaxation, increased diastolic filling pressure and left atrial dilatation [1,5,7,8]. Left atrial dilatation increases the risk of AFib [4,5] and the combination of impaired atrial contraction, LV hypertrophy and elevated LV filling pressure leads to diastolic and systolic dysfunction causing more symptoms and clinical deterioration [5,9]. There is a moderate overrepresentation of AFib in AS, indicating that AFib is related to the severity of AS [10] and possibly a sign of progressive deterioration of AS. Pre-operative AFib is a predictor of post-operative mortality and/or morbidity after aortic valve replacement [11-14], and longstanding AFib in AS is associated with increased risk of heart failure [10].

Echocardiographic assessment of AS in sinus rhythm (SR) is well documented; however, echocardiographic documentation of patients with AS and AFib is limited. The beat-to-beat variation in AFib complicates echocardiographic assessment and patients with AFib are often excluded from echocardiographic clinical trials. Clinical echocardiographic trials addressing the combination of AFib in AS are therefore needed.

We hypothesized that AFib is an adverse sign in AS in general, leading to impaired cardiac function as assessed by echocardiography and being an independent risk factor of mortality.

## Methods

### Study design

The study was designed as a single-center case-control study. Since 2005, all echocardiographic examinations at Gentofte University Hospital have been digitally stored. Patient data were collected from patient files and a local hospital database for patients undergoing invasive cardiac treatment or thoracic surgery.

### Study population

Echocardiographic examinations of hospitalized patients and out-patients at the Department of Cardiology, Gentofte University Hospital, are performed by dedicated sonographers according to a standardized protocol. All exams are digitally stored in a database on a central server. From this database all patients with AS and a standard transthoracic echocardiographic examination (n = 1,002) were selected. We found 117 patients diagnosed with both AS and AFib. 6 patients were excluded because of poor image quality and 8 patients were diagnosed with paroxysmal AFib, but were in SR at the time of echocardiography. The remaining 103 patients were included in the study as cases (AS and AFib at the time of echocardiography). After echo analysis of the 103 cases; 103 matching controls were found in the database. Matching was made by age, gender, and severity of AS. Some cases had >1 matching control in the database, in these circumstances the controls were randomly selected by lot.

### Outcome

Outcome was all cause mortality. Participants were followed until May 2011 or time of death, using the unique personal identification number in the Central Office of Civil Registration. Follow-up was 100% complete.

### Patient data

Height, weight, medications, symptoms, co-morbidities and cardiovascular risk factors were collected from a local database at the Department of Cardiology and the Department of Cardiothoracic Surgery, Gentofte University Hospital and from patient files. Body Surface Area (BSA) was calculated using the DuBois formula:

$$BSA\left(m^{2}\right)=0.20247\times Weight{\left(kg\right)}^{0.425}$$

### Echocardiographic analyses

Echocardiography was performed using Vivid 7 or Vivid E9 (General Electric Healthcare, Horten, Norway) between December 2005 and December 2010 and analyzed *de novo* by one person blinded to clinical information using EchoPAC PC version 108.1.12 (General Electric Healthcare, Horten, Norway). Most patients had more than one examination in the database. To ensure early inclusion and long follow-up the first digitally stored examination was included. Severity of AS were graded in mild, moderate and severe AS in agreement with current guidelines [15]. Maximum pressure gradient was calculated from the maximum jet velocity across the aortic valve and the left ventricular outflow tract (LVOT) using the modified Bernoulli equation. LVOT diameter was measured in mid-systole from the parasternal long-axis view. Aortic Valve Area (AVA) was calculated by the continuity equation [15]. Mild degrees of mitral and aortic regurgitation were evaluated using multiple views of color flow imaging measuring the origin, direction and size of the regurgitation jet. In suspicion of moderate or severe regurgitation; vena contracta width, pressure half-time (for aortic regurgitation) and if possible also regurgitant volume was calculated [16,17].

Heart rate was averaged from 15 heart cycles in AFib and 7 heart cycles in SR. To optimize echocardiographic assessment of LV function and to reduce the influence of beat-to-beat variation in AFib, means from two or more heart cycles were used.

LV dimensions were estimated from the parasternal long-axis view. LV mass was calculated using the Devereux formula [18] and indexed to BSA. The LV end-diastolic and end-systolic volumes and left ventricular ejection fraction (LVEF) were estimated using Simpson’s method in the apical four- and two-chamber view. End-systolic left atrial volume was calculated using the area-length method in the apical four- and two-chamber view.

Trans-mitral Early inflow (E) and Deceleration Time were obtained from pulsed wave Doppler in the apical four-chamber view. Pulmonary valve jet velocity was obtained using continuous wave Doppler from the parasternal short-axis view. Tricuspid valve regurgitation velocity was obtained using continuous wave Doppler from a modified apical four-chamber-view optimized for the right-sided chambers. Peak gradients for pulmonary valve and tricuspid valve regurgitation were calculated using the Bernoulli equation [15]. Right atrial pressure was estimated as normal (3 mmHg), intermediate (8 mmHg) and high (15 mmHg) from size and inspiratory response of the inferior vena cava in the subxiphoidal view [19]. Systolic pulmonary artery pressure was determined by adding the tricuspid valve regurgitation gradient and the estimated right atrial pressure. Peak early diastolic longitudinal mitral annular velocity (e’) was measured using pulsed wave tissue Doppler in the lateral mitral annulus in the apical four-chamber view. Iso-volumetric relaxation time were calculated using tissue Doppler M-mode of the anterior mitral leaflet in the apical four-chamber-view [20].

### Operative risk calculation

The operative mortality risk of aortic valve replacement was evaluated for each patient using European System for Cardiac Operative Risk Evaluation (EuroSCORE). Variable definitions were in accordance to the definitions listed on the EuroSCORE website ( http://www.euroscore.org/). To minimize the influence of low LVEF, EuroSCORE was also calculated without the influence of “LV dysfunction”.

### Statistical analyses

Continuous variables are presented as median and interquartile range, and categorical variables as number and percentages. Fisher’s exact test was used to compare categorical data and since many of the continuous variables were not normally distributed, Wilcoxon signed-rank test was used to compare continuous variables. Cumulative survival curves were established by the Kaplan-Meier method, and the curves were compared using the log-rank test. Cox proportional hazards regression models were used to examine the associations of AFib and baseline variables with the risk of mortality. All tests of significance were two-tailed. Statistical significance was defined as p < 0.05. SAS software (SAS System for Windows, release 9.2, SAS Institute Inc., Cary, NC, USA) was used to perform all statistical analyses.

## Results

### Study population

A total of 206 patients with AS (103 cases with AFib and 103 matched controls with SR at baseline) were included in the study. Population characteristics are summarized in Table 1. Five patients in the control group received Warfarin: indications were severe atherosclerosis, pulmonary emboli, apical aneurism with thrombus and congenital anti-thrombin deficiency. There was a trend for significant difference between the two groups regarding the presence symptoms (p = 0.08); the control group reported more frequently angina whereas dyspnea were more frequent in the case group (Table 1). EuroSCORE were lower in the case group, however, this difference was all explained by the difference in LVEF between the groups: when removing the influence of LV dysfunction from EuroSCORE, there were no significant differences between cases and controls (Table 1). There was a difference in surgery between the two groups; 59 controls and 45 cases underwent aortic valve replacement (p = 0.05).

**Table 1:** Population characteristics at baseline

	**CONTROLS**		**CASES**		**P-value**
	***Aortic stenosis & sinus rhythm***		***Aortic stenosis & atrial fibrillation***		
Age , years	78	(73–84)	78	(73–84)	0.96
Male gender, n (%)	70	(68)	70	(68)	1.00
Body Mass Index, kg/m^2^	25.7	(22.3–28.1)	25.5	(22.3–27.5)	0.65
Body Surface Area, m^2^	1.88	(1.76–2.06)	1.89	(1.75–2.06)	0.90
Heart rate (bpm)	68	(63–77)	81	(73–96)	<0.001
**Cardiovascular risk factors and co-morbidities**					
Hypertension, n (%)	57	(57)	55	(55)	0.78
Smoking (previous or current), n (%)	57	(64)	65	(72)	0.24
Peripheral vascular disease, n (%)	5	(5)	13	(16)	0.02
Diabetes (Type I or II), n (%)	22	(21)	25	(24)	0.62
Hypercholesterolemia, n (%)	47	(49)	46	(46)	0.63
Stroke, n (%)	15	(15)	14	(15)	0.94
Ischaemic heart disease, n (%)	27	(26)	34	(33)	0.29
Familiar disposition, n (%)	14	(18)	16	(22)	0.49
Chronic lung disease, n (%)	22	(21)	17	(17)	0.38
**Medication**					
β-blockers, n (%)	37	(41)	58	(58)	0.02
Calcium-antagonists, n (%)	21	(23)	22	(22)	0.83
ACE-inhibitors and/or Angiotensin-II-receptor-antagonists, n (%)	42	(47)	38	(38)	0.23
Diuretics (any kind), n (%)	46	(51)	65	(65)	0.05
Loop-diuretics, n (%)	19	(21)	51	(51)	<0.001
Thiazides, n (%)	27	(30)	15	(15)	0.013
Potassium-sparing diuretics, n (%)	6	(7)	9	(9)	0.55
Insuline, n (%)	11	(12)	3	(3)	0.02
Oral antidiabetics, n (%)	10	(11)	14	(14)	0.55
Statins, n (%)	51	(57)	48	(48)	0.23
Aspirin, n (%)	57	(63)	50	(50)	0.06
Inhaled Medications (glucocorticoids/anticholinergics/β2-agonists), n (%)	9	(10)	11	(11)	0.82
Warfarin, n (%)	5	(6)	59	(59)	<0.001
Digoxin, n (%)	0		45	(45)	<0.001
Amiodarone, n (%)	0		2	(2)	0.18
**Operative risk calculations**					
Additive EuroSCORE	8	(7–10)	9	(7–10)	0.05
Additive EuroSCORE without LV dysfunction	7	(6–9)	7	(6–9)	0.39
**Symptoms**					
Symptoms (angina pectoris, dyspnea, dizziness, syncope), n (%)	75	(77)	87	(86)	0.08
Angina pectoris, n (%)	43	(43)	28	(28)	0.02
Dyspnea, n (%)	59	(63)	80	(80)	0.008
Dizziness, n (%)	12	(13)	17	(17)	0.41
Syncope, n (%)	5	(5)	9	(9)	0.31

### Echocardiography

Results from the echocardiographic examinations are summarized in Table 2. The case group had a lower median LVEF and stroke volume, but there was no difference in cardiac output and cardiac index (Table 2). Aortic velocities and gradients were lower in the case group, but when AVA was calculated by the continuity equation no significant differences were found (Table 2). Cases and controls were matched on the severity of AS (p = 1.00). No differences with regard to aortic regurgitation (p = 0.69) were found; by contrast the severity of mitral regurgitation was distributed differently between the two groups (p = 0.002), cases had a more severe degree of mitral regurgitation than the controls: None (15% vs. 6%), trivial (46% vs. 28%), mild (36 % vs. 59%), moderate (2% vs. 6%), and severe (1% vs. 1%) mitral regurgitation.

**Table 2:** Echocardiograpic findings

	**CONTROLS**		**CASES**		**P-value**
	***Aortic stenosis & sinus rhythm***		***Aortic stenosis & atrialfibrillation***		
	**Median**	**(IQR)**	**Median**	**(IQR)**	
**Left ventricle**					
LVEF (%)	49	(41–56)	42	(33–51)	<0.001
LVEDV (ml)	112	(91–144)	119	(87–148)	0.86
LVESV (ml)	58	(40–75)	67	(43–91)	0.15
Stroke volume (ml)	55	(44–68)	47	(40–57)	0.004
Cardiac output (l)	3.8	(3.0–4.7)	4.0	(3.3–4.8)	0.29
Cardiac index (l/m^2^)	2.1	(1.6–2.5)	2.1	(1.7–2.5)	0.63
LVM (g)	159	(125–208)	172	(133–228)	0.08
LVMI (g/m^2^)	87	(71–109)	94	(77–119)	0.17
**Left atrial dimensions and volumes**					
LAV (ml)	82	(68–101)	92	(80–126)	0.006
LAV index (ml/m^2^)	46	(38–54)	48	(40–59)	0.11
LAD (cm)	3.9	(3.5–4.4)	4.7	(4.2–5.3)	<0.001
**Aortic valve velocities**					
AV jet velocity (m/s)	4.0	(3.3–4.6)	3.3	(2.8–4.1)	0.004
AV peak gradient (mmHg)	59	(39–79)	41	(28–64)	<0.001
AV mean gradient (mmHg)	33	(21–43)	22	(15–38)	0.001
AVA (cm^2^)	0.88	(0.70–1.10)	0.85	(0.65–1.07)	0.38
**Mitral inflow and diastolic measures**					
E (m/s)	0.77	(0.64–0.96)	1.04	(0.87–1.25)	<0.001
DT (ms)	203	(148–267)	149	(123–189)	<0.001
IVRT (ms)	98	(80–106)	77	(65–94)	<0.001
e’ (lateral mitral annulus) (cm/s)	0.05	(0.04–0.07)	0.09	(0.08–0.12)	<0.001
E/e’	14.2	(10.9–19.0)	11.1	(8.07–15.7)	<0.001
**Pulmonary pressure measurements**					
TRmax (mmHg)	29	(24–35)	32	(26–39)	0.047
RA pressure (mmHg)	3	(3–3)	3	(3–8)	0.003
SPAP (mmHg)	32	(26–41)	36	(29–46)	0.028

*AV* Aortic valve, *AVA* Aortic valve area, DT = Mitral valve decerleration time, E = Mitral valve peak early diastolic inflow, e’ = Peak early diastolic velocity by pulsed tissue doppler, *IQR* Interquartile range, *IVRT* Isovolumetric relaxation time, *LAD* Left atrial diameter, *LAV* Left atrial end-systolic volume, *LVEDV* Left ventricular end-diastolic volume, *LVEF* Left ventricular ejection fraction, *LVESV* Left ventricular end-systolic volume, *LVM* Left ventricular mass, *LVMI* Left ventricular mass index, *n* numbers, RA *pressure* Estimated right atrial pressure, *SPAP* Estimated systolic pulmonary artery pressure, *TRmax* Tricuspid valve regurgitant peak gradient.

### Mortality

**Figure 1:**
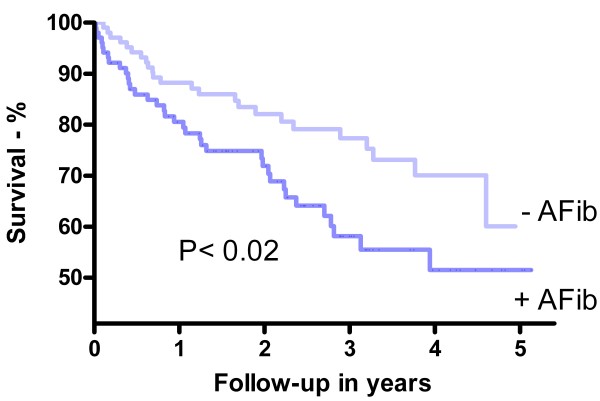
**Kaplan - Meier survival curves.** Shown is the cumulative survival in patients with aortic stenosis and atrial fibrillation (+AFib), compared to patients with aortic stenosis in sinus rhythm (-AFib). The curves are compared using the log-rank test.

During a median follow-up of 2.3 years (IQR: 1.2–3.6), 70 (34%) patients died: 42 patients with AS and AFib and 28 patients with AS and SR (p < 0.02). Patients with AS and AFib had a significantly higher risk of death compared to controls (Figure 1).

Severe AS was present in nearly 50% of the patients at baseline, but the mortality risk remained significantly higher in cases after adjustment for severe AS (hazard ratio 1.74, 95% confidence interval: 1.07–2.83; p < 0.03). No significant interaction was found between severity of AS and AFib on the risk of mortality (p = 0.62).

Significant differences in the echocardiographic parameters were found between the case and control group (Table 2) however AFib remained significantly associated with mortality after adjustments for conventional echocardiographic parameters of forward and backward failure (Table 3). Similarly, AFib remained significantly associated with mortality (hazard ratio 1.78, 95% confidence interval: 1.10–2.91; p < 0.02) after adjustment for mitral regurgitation (p = 0.80).

**Table 3:** Atrial fibrillation as a predictor of mortality by Cox proportional hazard regression models

	**Hazard ratio**	**P-value**
	**(95% CI)**	
Model	1.94 (1.19-3.15)	P < 0.01
Model + LVEF	1.75 (1.05-2.92)	P < 0.03
Model + LAV	1.84 (1.04-3.26)	P < 0.04
Model + TRmax	2.06 (1.20-3.56)	P < 0.01
Model + RA pressure	2.21 (1.19-4.12)	P < 0.02
Model + SPAP	2.37 (1.24-4.52)	P < 0.02
Model + E/e’	2.80 (1.66-4.72)	P < 0.001
Model + LVEF + LAV + TRmax + SPAP + E/e’	2.72 (1.12-6.61)	P < 0.03

Model = Adjusted for age, gender and aortic stenosis severity.

*CI* Confidence interval, *LAV* Left atrial end-systolic volume, *LVEF* Left ventricular ejection fraction, *RA pressure* Estimated right atrial pressure, *SPAP* Estimated systolic pulmonary artery pressure, *TRmax* Tricuspid valve regurgitant peak gradient.

## Discussion

### Atrial fibrillation predicts mortality in aortic stenosis

In this study we analyzed echocardiographic examinations from 103 patients with AS and AFib and compared them with 103 controls (with AS but and SR) matched on age, gender and severity of AS. We found that patients with coexistence of AS and AFib had significantly higher risk of death compared to controls (Figure 1). This difference remained after adjustment for LVEF, left atrial volume, maximal tricuspid valve regurgitation, estimated right atrial pressure, estimated systolic pulmonary artery pressure and E/e’ (Table 3). A few studies have described an increased mortality in patients with AS and AFib undergoing surgical intervention [11-14], and one study based on a selected population have found an increased risk of heart failure and non-hemorragic stroke in AS and AFib. The same study also found that new-onset AFib was associated with cardiac decompensation [10]. More studies describing mortality and morbidity before surgical intervention (or when surgical intervention is not an option) are needed. It seems as if this patient group has become “scientifically neglected”. The prognostic effect of AFib in heart failure is well documented in clinical trials; in a meta-analysis AFib was associated with total mortality in both randomized controlled trials and observational studies [2] and another study found an increased risk of death after adjusting for pre-existing cardiovascular conditions related to AFib [4].

We found no significant interaction between severity of AS and AFib on the risk of mortality, emphasizing that the presence of AFib in AS worsen prognosis regardless of the severity of the AS. Thus, AFib predicts bad outcome not only in patients with severe AS but also in patients with mild or moderate AS. The finding of AFib being an independent risk factor in AS is an important original discovery since the diagnosis of AFib from an electrocardiogram (ECG) is far more accessible than evaluation of cardiac function by echocardiography Additional file 1: Video 1 and Additional file 2: Video 2.

### Echocardiographic differences between sinus rhythm and atrial fibrillation

**Figure 2:**
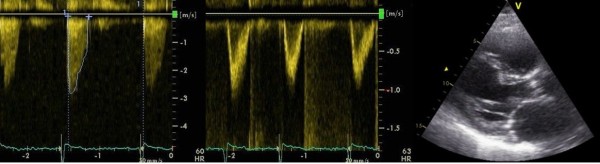
**Calculation of the Aortic Valve Area by the continuity equation, patient in atrial fibrillation.** Aortic jet velocity, left ventricular outflow velocity and diameter of the left ventricular outflow tract. Male patient aged 77 with severe aortic stenosis, matched to the patient in Figure 3. Aortic valve area (0.80 cm^2^). Ejection fraction (31%). Cardiac Index (1.80 l/m^2^). Stroke volume (50 ml).

**Figure 3:**
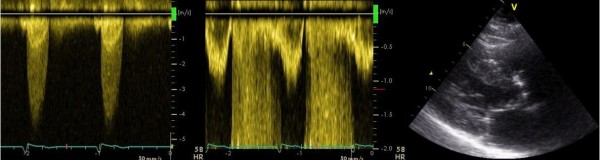
**Calculation of the Aortic Valve Area by the continuity equation, patient in sinus rhythm.** Aortic jet velocity, left ventricular outflow velocity and diameter of the left ventricular outflow tract. Male patient aged 77 with severe aortic stenosis, matched to the patient in Figure 2. Aortic valve area (0.88 cm^2^). Ejection fraction (43%). Cardiac Index (1.93 l/m^2^). Stroke volume (63 ml).

We observed lower LVEF and stroke volume in the group with AFib; however, the group with AFib also had higher heart rate but no differences in cardiac output or cardiac index when compared to the group with SR. Similarly, the group with AFib had lower aortic jet velocity and gradients but when evaluating the aortic velocities to the velocities in LVOT using the continuity equation no differences were found with regard to AVA (Table 2). Severity of AS is determined using the aortic jet velocity, mean gradient and AVA [15] and since trans-valvular velocities are lower in AFib, patients with AFib might be graded with a less severe degree of AS. There was no difference in AVA, indicating that velocities and gradients should be used in the continuity equation for calculation of the AVA and not for grading of AS severity in AFib (Figures 2 and 3)*.*

Diastolic measurements and interpretation of diastolic dysfunction should always be used with careful attention in atrial fibrillation. Absence of atrial contraction in AFib makes it necessary to interpret diastolic filling from parameters independent of atrial contraction; such as E/e’, Iso-volumetric relaxation time and deceleration time. However, those parameters will always be affected in the presence of AFib. Loss of atrial contraction and only one diastolic filling phase causes higher e’ and consequently low E/e’. Both iso-volumetric relaxation time and deceleration time are dependent of heart rate. High heart rate causes short iso-volumetric relaxation time and paradoxically short deceleration time [21,22].

### Echocardiographic difficulties in atrial fibrillation

Echocardiographic assessment of AS during AFib is difficult and requires reflection. Myocardial contractility depends on the preceding (R-R_1_) and pre-preceding (R-R_2_) R-R interval, which is constantly changing in AFib. (R-R_1_)/(R-R_2_) = 1 should represent the average value of contractility for all cardiac cycles in AFib and should therefore be used [23]. In the clinical setting the two preceding R-R intervals should have identical R-R intervals. We used means from as many cycles as possible, usually 2–7 cycles, and tried to use cycles where the preceding and pre-preceding cycles were visible, but this was unfortunately not always possible. In general short R-R intervals cause poor relaxation and impaired diastolic filling, whereas longer R-R intervals favors relaxation and proper diastolic filling [5]. Preceding R-R intervals between 875–935 ms should assure sufficient diastolic filling. Dubery *et al.* tried to find the optimal number of beats for Doppler measurements in AFib. They found that a mean of 13 beats was required to achieve an estimation of cardiac output with a variability of less than 2% [24]. The Echocardiographic assessment of valve stenosis: EAE/ASE recommendations for clinical practice suggests measurement from a minimum of five cardiac cycles if AFib in mitral stenosis and tricuspid stenosis [15].

We suggest that; for optimal echocardiographic assessment in AFib, importance should be put on optimizing the calculation of the AVA by the continuity equation. One should, as always, pay attention to the technical details for accurate Doppler jet velocity curves. When interpreting Doppler jet velocities over valves; any deviation from the parallel angle of the blood flow will result in underestimation of the jet velocity and should be avoided. When grading the severity, most importance should be put on the AVA. Heart rate should be 60–70 bpm and R-R intervals used in measurement should not differ from each other.

### Timing of surgery in atrial fibrillation

Timing of surgery in AS should be based on both clinical and echocardiographic assessment [25-27]. However, both clinical and echocardiographic assessment might be complicated in AFib. The natural history of AS is characterized by a phase with increased obstruction and adaptive mechanisms in the ventricle called the “latent” period. Once symptoms occur, the clinical outcome is extremely poor, with two-year survival rates <50% [3,9,27,28]. Symptoms of AS might be dyspnea, angina pectoris, dizziness or syncope, which correlates very well to the symptoms of AFib and it might be difficult or even impossible to assess the underlying real etiology of symptoms [29]. Not surprisingly; AFib is associated with missed diagnosis of severe AS, despite systolic murmur and symptoms of angina pectoris [30]. Langanay *et al.* found AFib to be predictive for an increased operative risk and suggests early referral of patients for surgery, before progression of disease and onset of cardiac dysfunction [14]. Most guidelines today recommend surgery of symptomatic patients with severe AS, which complicates decision making and threatens adequate treatment for patient with AFib. There are a few circumstances where also asymptomatic patients with AS are recommended surgery. These are patients with severe or moderate AS undergoing coronary artery bypass surgery or surgery of the ascending aorta, severe AS and systolic LV dysfunction with LVEF <50%, severe AS and abnormal exercise testing (symptoms during exercise, fall in blood pressure below baseline, complex ventricular arrhythmias), moderate-to-severe and severe AS with rapid progression of peak velocity, severe AS and excessive LV hypertrophy not due to hypertension and low gradient (<40 mmHg) AS and LV dysfunction with or without contractile reserve. The decision to operate on asymptomatic patients requires careful weighting of benefits against risks and should only be performed in selected patients at low operative risk [25,26]. The clinical setting contains many dimensions and the etiologies of symptoms in our population are most likely a combination of both AS and AFib.

The combination of doubts regarding etiology of symptoms and echocardiographic difficulties in AFib might increase the risk of surgical delay, with the risk that patients develop high surgical risk, heart failure and becomes in-operable with poor chance of survival. In our study, aortic valve replacement was more frequent in the control group, despite the fact that cases and controls were matched according to age, sex and AS severity. Withholding of aortic valve replacement may well be an intermediate component cause of atrial fibrillation predicting mortality, which emphasizes the importance of an aggressive approach towards surgery in patients with AS and atrial fibrillation. Some other clinical trials have found AFib to be a predictor of per-operative and post-operative mortality in patients receiving AVR [11-14]. However, in an Italian multi-center study, AFib was not a predictor of mortality after Transcatheter Aortic Valve Implantation [31] and in a smaller study no difference in prognosis between AFib and SR after TAVI was observed, despite a higher surgical risk in the group with AFib [32].

As incidences of both AFib and AS increases with age and as the population is aging, more patients with both conditions must be expected in the future; clinical trials addressing the combination of AFib and AS and the echocardiographic assessment are needed.

### Potential limitations

Our data was retrospectively collected from the echocardiography database, patient files, discharge letters and the local database for patients undergoing cardiovascular intervention or thoracic surgery. Some data were unfortunately not accessible. There were 25 cardiovascular deaths in the case-group and 15 cardiovascular deaths in the control-group. Cause of death was unfortunately not available for 12 patients and statistics regarding impact on cardiovascular has therefore not been included in the manuscript.

We did not distinguish between paroxysmal and permanent AFib, therefore some of the patients in the case group might have had only a single episode of AFib (during the echocardiographic examination). There is also a risk that some of the patients in the control group might have had unknown episodes of paroxysmal AFib. However, this would only dilute the effect of AFib.

The study was performed at a tertiary referral center. Many patients were followed elsewhere and referred to our department because of deterioration in clinical status and/or evaluation for surgery. The population might not be representative of the AS patient and there might be a false shortening of follow-up time.

The study cohort is composed of exclusively Caucasians, which may limit the generalizability of our findings to other ethnic groups.

## Conclusion

AFib is an independent risk factor of mortality in patients with AS and AFib predicts bad outcome in patients with not only severe AS but also in mild or moderate AS. At the clinical follow-up of the patient with AS and AFib both conditions must be in mind when evaluating symptoms and when quantifying the severity of AS.

## Abbreviations

AS: Aortic Stenosis; AVA: Aortic Valve Area; AFib: Atrial Fibrillation; BSA: Body Surface Area; EuroSCORE: European System for Cardiac Operative Risk Evaluation; LV: Left Ventricular; LVOT: Left Ventricular Outflow Tract; LVEF: Left Ventricular Ejection Fraction; SR: Sinus Rhythm.

## Competing interests

The authors declare that they have no competing interests.

## Authors’ contributions

CBK performed the analysis of the echocardiographic material, interpreted the data and drafted the manuscript. RM performed the statistical analysis. RM, JSJ, PS and HGC added clinical discussion to the manuscript and contributed to analysis, interpretation and presentation of data. All authors read and approved the final manuscript.

## Supplementary Material

Additional file 1 **Video 1.** Atrial fibrillation. Parasternal long-axis view.Case and control (77 year, male, severe aortic stenosis). The same patientsused as example in Figures 1 and 2. The patient with atrial fibrillationdied of heart failure after 2.2 years of follow up and the patient in sinusrhythm was still alive after maximal follow up of 4.3 years. Click here for file

Additional file 2 **Video 2.** Sinus rhythm. Parasternal long-axis view.Case and control (77 year, male, severe aortic stenosis). The same patientsused as example in Figures 1 and 2. The patient with atrial fibrillationdied of heart failure after 2.2 years of follow up and the patient in sinusrhythm was still alive after maximal follow up of 4.3 years. Click here for file
